# Phylattrin, a New Cytotoxic Xanthone from *Calophyllum soulattri*

**DOI:** 10.3390/molecules17078303

**Published:** 2012-07-10

**Authors:** Siau Hui Mah, Gwendoline Cheng Lian Ee, Soek Sin Teh, Mawardi Rahmani, Yang Mooi Lim, Rusea Go

**Affiliations:** 1 Department of Chemistry, Faculty of Science, Universiti Putra Malaysia, 43400 UPM Serdang, Selangor, Malaysia; 2 Faculty of Medicine and Health Science, Universiti Tunku Abdul Rahman, 43000 Kajang, Selangor, Malaysia; 3 Department of Biology, Faculty of Science, Universiti Putra Malaysia, 43400 UPM Serdang, Selangor, Malaysia

**Keywords:** *Calophyllum soulattri*, Clusiaceae, cytotoxic, phylattrin, xanthone

## Abstract

Our continuing studies on secondary metabolites from the stem bark of *Calophyllum soulattri* has led to the isolation of another new diprenylated xanthone, phylattrin (**1**), in addition to five other xanthones and two common sterols. The xanthones are soulattrin (**2**), caloxanthone C (**3**), macluraxanthone (**4**), brasixanthone B (**5**) and trapezifolixanthone (**6**) while the sterols are stigmasterol (**7**) and *β*-sitosterol (**8**). The structures of these compounds were determined on the basis of spectroscopic analyses such as 1D and 2D-NMR, HRESIMS, IR and UV. Compounds **1**–**7** exhibited moderate cytotoxic activities against SNU-1, HeLa, Hep G2, NCI-H23, K562, Raji, LS174T, IMR-32 and SK-MEL-28 cells.

## 1. Introduction

*Calophyllum* belongs to the Clusiaceae family and is mainly distributed in tropical areas around the World, primarily in the Indo-Pacific region. It has many local names such as “bintangor” in Asia and “tamanu” in Hawaii. *Calophyllum* is well known for its biological activities which are the result of the presence of a variety of secondary metabolites. Xanthones [1,2,3,4], coumarins [5], triterpenoids [6] and flavonoids [7,8] are examples of the most reported secondary metabolites. Among all the biological activities, anti-HIV [9,10] and anti-cancer [11] were the most reported. Besides, some of these compounds are widely used in industry such as antiseptics, astringents, diuretics and purgatives [12]. This present paper reports the isolation and structural elucidation of a new compound, phylattrin (**1**) from the stem bark of *Calophyllum soulattri*. The cytotoxicities of the new compound and seven known compounds isolated at the same time will also be reported.

## 2. Results and Discussion

**Figure 1:**
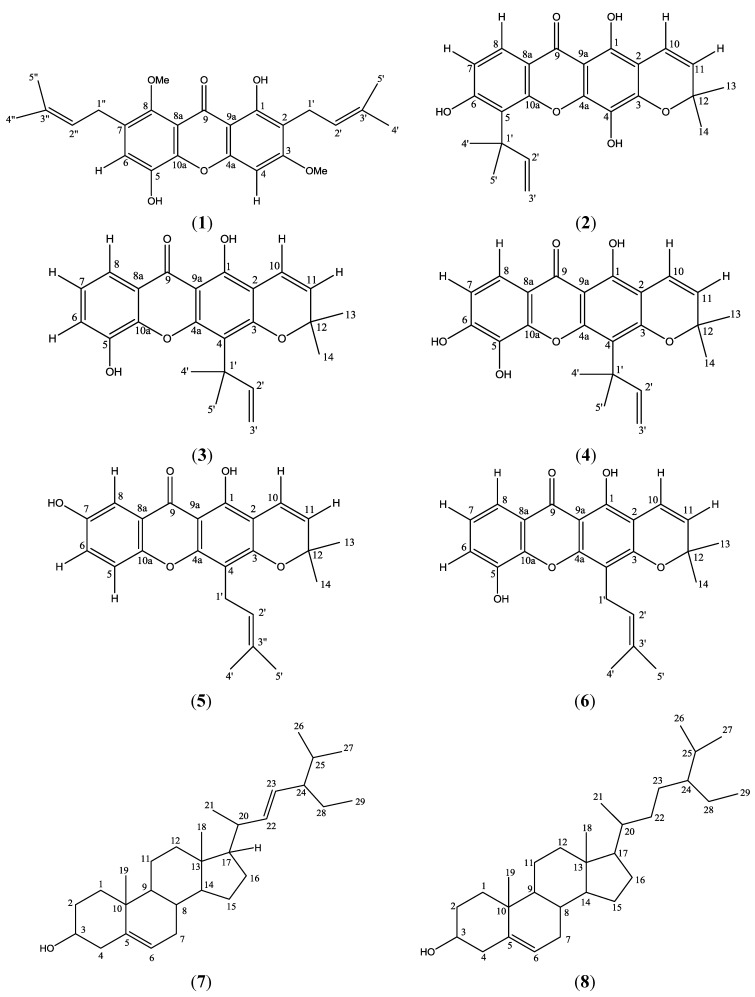
Structures of phylattrin (**1**), soulattrin (**2**), caloxanthone C (**3**), macluraxanthone (**4**), brasixanthone B (**5**), trapezifolixanthone (**6**), stigmasterol (**7**) and *β*-sitosterol (**8**).

The stem bark of *Calophyllum soulattri* was ground and extracted with *n*-hexane and dichloromethane. Chromatographic techniques were applied on both vacuum dried extracts for separation and purification purposes. The *n*-hexane extract gave one sterol, *β*-sitosterol (**8**), while the dichloromethane extract afforded five known xanthones: soulattrin (**2**), caloxanthone C (**3**), macluraxanthone (**4**), brasixanthone B (**5**) and trapezifolixanthone (**6**) and another common sterol, stigmasterol (**7**). All the compounds were identified by comparison of their spectral data with literature values [4,5,13,14,15]. See Figure 1.

Meanwhile, the dichloromethane extract contributed a new xanthone derivative, phylattrin (**1**). It was isolated as a yellowish crystalline material which gave a melting point of 173–174 °C. The EIMS spectra gave a molecular ion peak at *m*/*z* 424, implying the molecular formula C_25_H_28_O_6_. The FTIR absorptions and UV spectrum suggested a xanthone moiety [14].

The ^1^H-NMR spectrum of **1** showed the presence of a chelated OH signal (*δ* 13.41). ^13^C-NMR and DEPT spectra supported the existence of a carbonyl carbon at *δ* 182.0 along with twelve quaternary carbons. The carbon signals at *δ* 137.3, 143.0, 155.3, 155.7, 159.5 and 163.5 are for the six oxygenated carbons while the non-oxygenated quaternary carbons resonated at *δ* 103.9, 111.5, 112.2, 131.8 × 2 and 137.3. Besides this, four methine carbon signals (*δ* 88.9, 101.8, 122.4 and 123.4), two methylene (*δ* 21.4 and 26.5) and four methyl carbon signals (*δ* 17.8, 18.2, 25.9 × 2) and two methoxyl signals (*δ* 55.9 and 61.7) were also observed in the ^13^C-NMR and DEPT spectra. 

**Figure 2:**
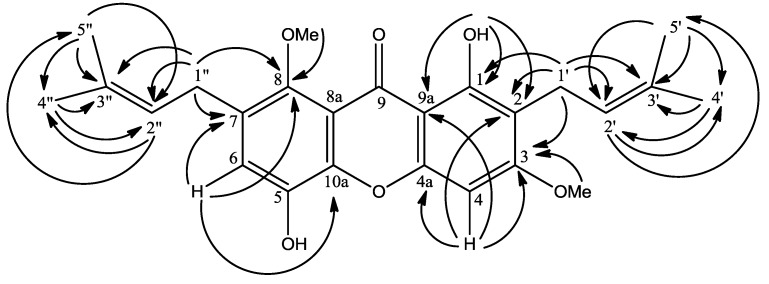
HMBC ^2^*J* and ^3^*J* correlations between ^1^H and ^13^C in **1**.

The chelated OH signal gave HMBC cross peaks with *δ* 103.9 (C-9a, ^3^*J*), 111.5 (C-2, ^3^*J*) and 159.5 (C-1, ^2^*J*) allowing its placement at C-1. Besides this, two sharp singlets were observed in the methoxyl region at *δ* 3.80 (8-OCH_3_) and 3.87 (3-OCH_3_) in the ^1^H-NMR spectrum. These singlets were attached to C-8 and C-3, which was deduced by long range HMBC correlations of *δ* 3.80 and 3.87 with *δ* 143.0 (C-8) and 163.5 (C-3) respectively (Figure 2). The remaining two sharp singlets represented two lone aromatic protons in **1**. The H-4 signal was correlated to *δ* 103.9 (C-9a), 111.5 (C-2), 155.7 (C-4a) and 163.5 (C-3) while H-6 correlated to 112.2 (C-7), 143.0 (C-8) and 155.3 (C-10a). Hence these singlets were assigned to the lone protons at C-4 and C-6 respectively.

The ^1^H-NMR spectrum of **1** revealed the presence of two prenyl moieties. Two pairs of vinylic signals were observed at *δ* 3.35 (*d*, 2H, *J =* 7.0 Hz) and 5.34 (*t*, 1H, *J =* 7.0 Hz) for H-1′ and H-2′ and *δ* 4.09 (*d*, 2H, *J =* 5.9 Hz) and 5.24 (*t*, 1H, *J =* 5.9 Hz) for H-1′′ and H-2′′. Meanwhile, two pairs of methyl signals appeared at *δ* 1.67 (*s*, 3H, H-4′) and 1.79 (*s*, 3H, H-5′) and 1.69 (*s*, 3H, H-4′′) and 1.83 (*s*, 3H, H-5′′). The doublet signal at *δ* 3.35 gave cross peaks to the carbon signal at *δ* 111.5 (C-2, ^2^*J*), 159.5 (C-1, ^3^*J*) and 163.5 (C-3, ^3^*J*) indicating the prenyl group to be directly attached to C-2. Also, another doublet signal at *δ* 4.09 (*d*, 2H, H-1′′) showed connectivity to *δ* 112.2 (C-7, ^2^*J*) and 143.0 (C-8, ^3^*J*) allowing us to link this second prenyl moiety directly to C-7′. However, the NMR chemical shifts data for C-4′ and C-5′ and C-4′′ and C-5′′ were not unambiguously assigned as they are interchangeable.

On the basis of the above interpretation of the 1D and 2D-NMR data, the structure of compound **1** was elucidated to be 1,5-dihydroxy-3,8-dimethoxy-2-(3′,3′-dimethyl-2′-propenyl)-7-(3′′,3′′-dimethyl-2′′-propenyl)-xanthone and named phylattrin (**1**). 

Compounds **1**–**8** were evaluated for their cytotoxic activities against several human cancer cell lines *in vitro* using the MTT method. The tested cell lines were SNU-1 (stomach), HeLa (cervical), Hep G2 (liver), NCI-H23 (lung), K562 (leukemia), Raji (lymphoma), LS174T (colon), IMR-32 (neuroblastoma) and SK-MEL-28 (skin) cells. Compounds **1**–**7** showed moderate cytotoxic activities against some of these cell lines, with moderate IC_50_ values, shown in Table 1. Compounds **2** and **4** gave IC_50_ values of 1.98 and 4.95 µM, respectively, against the stomach cancer (SNU-1) cell line. Meanwhile, compounds **1**, **2**, **3**, **4** and **6** indicated cytotoxic activities against the HeLa (cervical) cell line with IC_50_ values of 9.20, 2.77, 6.88, 6.95 and 7.57 µM, respectively. Compounds **2** and **4** also indicated cytotoxicity in the tests for the NCI-H23 and Raji cell lines. Meanwhile, in the cytotoxic assays for the Hep G2 and LS174T cell lines only one compound gave activity. These are compound **3** for the Hep G2 cell line (IC_50_ value 6.22 µM) and compound **2** for the LS174T cell line (IC_50_ value 3.17 µM). Compounds **1** and **7** gave moderate cytotoxic activities against the SK-MEL-28 cell line (IC_50_ values 1.45 and 9.47 µM respectively), while compounds **2** and **4** gave reasonably good IC_50_ values against the IMR-32 cell line (IC_50_ values 0.69 and 4.95 µM respectively). Phylattrin (**1**) is cytotoxic for stomach, cervical and lung cancer while soulattrin (**2**) is cytotoxic for all the cancers tested which are stomach, cervical, lung, liver, leukemia, lymphoma, colon, skin and neuroblastoma cancer. 

**Table 1:** Cytotoxicity (IC_50_, μM) of compounds 1–8 against SNU-1 (stomach), HeLa (cervix), NCI-H23 (lung), Hep G2 (liver), K562 (leukemia), Raji (lymphoma), LS174T (colon), SK-MEL-28 (skin) and IMR-32 (neuroblastoma) cells.

Compounds	IC_50_ (μM) *								
	SNU-1	HeLa	NCI-H23	Hep G2	K562	Raji	LS174T	SK-MEL-28	IMR-32
1	9.79 ± 0.23	9.20 ± 0.83	10.45 ± 0.58	20.64 ± 0.86	22.10 ± 0.61	11.67 ± 0.16	11.04 ± 1.08	11.04 ± 1.24	18.42 ± 1.06
2	1.98 ± 0.47	2.77 ± 0.59	2.64 ± 0.45	12.87 ± 1.03	2.23 ± 0.13	2.56 ± 0.80	3.17 ± 0.55	1.45 ± 1.04	0.69 ± 0.91
3	24.79 ± 0.78	6.88 ± 0.91	15.42 ± 0.83	6.22 ± 0.75	18.20 ± 0.76	15.50 ± 1.14	28.94 ± 0.57	>100.00	18.60 ± 0.59
4	4.95 ± 0.85	6.95 ± 0.47	4.62 ± 0.38	11.12 ± 0.72	5.28 ± 0.22	4.44 ± 0.50	15.08 ± 0.94	39.64 ± 0.74	4.95 ± 1.20
5	20.66 ± 0.43	>100.00	44.10 ± 1.19	24.81 ± 0.45	31.00 ± 0.21	26.16 ± 0.47	36.38 ± 0.37	28.92 ± 0.18	92.59 ± 1.00
6	15.71 ± 0.28	7.57 ± 0.44	82.67 ± 0.78	27.54 ± 1.48	>100.00	20.66 ± 1.29	49.60 ± 0.71	26.85 ± 0.63	>100.00
7	>100.00	>100.00	>100.00	>100.00	>100.00	0.41 ± 0.87	>100.00	9.47 ± 0.84	>100.00
8	>100.00	>100.00	>100.00	>100.00	>100.00	>100.00	>100.00	>100.00	>100.00
Kaempferol **	38.22 ± 0.08	17.48 ± 1.13	65.56 ± 0.21	>100.00	>100.00	43.71 ± 0.80	>100.00	76.15 ± 0.86	>100.00
Quercetin **	20.86 ± 1.01	26.49 ± 1.00	57.95 ± 0.31	17.25 ± 0.95	32.75 ± 1.20	6.89 ± 0.40	>100.00	72.45 ± 0.84	>100.00

* The data shown are means ± SEM of three independent experiments; ** Positive control.

## 3. Experimental

### 3.1. General

EIMS were recorded on a Shimadzu GC-MS model QP2010 Plus spectrophotometer. NMR spectra were obtained using a JEOL FT-NMR 500 MHz spectrophotometer using tetramethylsilane (TMS) as internal standard. Ultraviolet spectra were recorded in EtOH on a Shimadzu UV-160A, UV-Visible Recording Spectrophotometer. Infrared spectra were measured using the universal attenuated total reflection (UATR) technique on a Perkin-Elmer 100 Series FT-IR spectrometer. Melting points were measured using a Leica Galen III microscope, equipped with a Testo 720 temperature recorder.

### 3.2. Plant Material

The stem bark of *Calophyllum soulattri* was collected from the Sri Aman district in Sarawak, Malaysia by Prof. Dr. Jegak Uli. This plant was identified by Dr. Rusea Go from the Department of Biology, Faculty of Science, Universiti Putra Malaysia where a voucher specimen was deposited.

### 3.3. Extraction and Isolation

Approximately 1 kg of air-dried stem bark of *Calophyllum soulattri* was ground into a fine powder and extracted successively in a Soxhlet apparatus with *n*-hexane and dichloromethane for 24 h. The extracts were evaporated to dryness under vacuum to give 101.2 g of hexane extract and 15.3 g of dichloromethane extract. Part of each extract was subjected to column chromatography over silica gel and eluted with a stepwise gradient system using *n*-hexane, dichloromethane, ethyl acetate and methanol. Further purifications of the hexane extract afforded *β*-sitosterol (**8**, 11 mg). Meanwhile, purification of the dichloromethane extract afforded the new xanthone, phylattrin (**1**, 67 mg), soulattrin (**2**, 7 mg), caloxanthone C (**3**, 14 mg), macluraxanthone (**4**, 6 mg), brasixanthone B (**5**, 21 mg), trapezifolixanthone (**6**, 10 mg) and stigmasterol (**7**, 22 mg). Compound **1** was obtained from the mixture of *n*-hexane–chloroform (1:9) eluate and followed by further purifications using a chromatotron and eluting with a chloroform–methanol (4.9:0.1) mixture.

*Phylattrin* (**1**) Yellow crystal; m.p. 173–174 °C; UV (EtOH) λ_max_ nm (log ε): 352 (5.48), 317 (5.43), 260 (5.34), 244 (5.32), 213 (5.26); IR ν_max_ cm^−1^: 3454, 2922, 1738, 1639, 1602, 1462; EIMS *m*/*z* (rel. int.): 424 [M^+^] (64), 381 (20), 369 (33), 368 (32), 354 (22), 353 (100), 337 (17), 335 (18), 325 (17), 299 (21); ^1^H-NMR (500 MHz, CDCl_3_): *δ* 13.41 (OH-1, s), *δ* 6.82 (1H, *s*, H-6), 6.33 (1H, *s*, H-4), 5.34 (1H, *t*, *J =* 7.0 Hz, H-2′), 5.24 (1H, *t*, *J =* 5.9 Hz, H-2′′), 4.09 (2H, *d*, *J =* 5.9 Hz, H-1′′), 3.87 (3H, *s*, 3-OCH_3_), 3.80 (3H, *s*, 8-OCH_3_), *δ* 3.35 (2H, *d*, *J =* 7.0 Hz, H-1′), 1.83 (3H, *s*, H-5′′), 1.79 (3H, *s*, H-5′), 1.69 (3H, *s*, H-4′′), 1.67 (3H, *s*, H-4′); ^13^C-NMR (125 MHz, CDCl_3_): *δ* 182.0 (C-9), 163.5 (C-3), 159.5 (C-1), 155.7 (C-4a), 155.3 (C-10a), 143.0 (C-8), 137.3 (C-5 & C-8a), 131.8 (C-3′ & C-3′′), 123.4 (C-2′′), 122.4 (C-2′), 112.2 (C-7), 111.5 (C-2), 103.9 (C-9a), 101.8 (C-6), 88.9 (C-4), 61.7 (8-OCH_3_), 55.9 (3-OCH_3_), 26.5 (C-1′′), 25.9 (C-4′ & C-4′′ or C-5′ & C-5′′), 21.4 (C-1′), 18.2 (C-4′ or C-5′), 17.8 (C-4′′ or C-5′′).

*Soulattrin* (**2**): Yellow crystals; m.p. 180–181 °C (Lit. 180–181 °C [4]); spectral data are consistent with published data [4].

*Caloxanthone C* (**3**): Yellow needles; m.p. 210–212 °C (Lit. 217 °C [14]); spectral data are consistent with literature [14].

*Macluraxanthone* (**4**): Yellow needles; m.p. 181–182 ºC (Lit. 170–172 °C [13]); spectral data are consistent with literature [13].

*Brasixanthone B* (**5**): Yellow crystal; m.p. 227–229 °C (Lit. 227–229 °C [16]); Spectral data are consistent with published data [16].

*Trapezifolixanthone* (**6**): Yellow crystal; m.p. 171–172 °C (Lit. 171–172 °C [17]); spectral data are consistent with published data [17].

*Stigmasterol* (**7**): White needles; m.p. 155–157 °C (Lit. 168–169 °C [15]); spectral data are consistent with literature [15].

*β-sitosterol* (**8**): White needles, m.p. 135–136 °C (Lit. 136–138 °C [15]). Spectral data are consistent with literature [15].

### 3.4. Cytotoxicity (MTT Assay)

The 3-(4,5-dimethylthiazol-2-yl)-2,5-diphenyltetrazolium bromide (MTT) assay was performed according to a previously described method [18]. The tests were performed in a sterile 96-well flat bottom plate. Stock solutions of each pure compound were prepared by dissolving them in DMSO to a concentration of 20 mg/mL. A six-point serial dilution was developed to obtain six different sub-stocks with different concentrations. For suspension cells, concentrations needed were 50.00, 25.00, 12.50, 6.25, 3.13 and 1.56 μg/mL. Meanwhile, 100.00, 50.00, 25.00, 12.50, 6.25 and 3.13 μg/mL were the essential concentrations for anchorage-dependant cells. Each pure compound was tested in triplicate together with the controls.

After 72 h incubation at 37 °C and 5% of CO_2_, MTT solution (20 μL) was added into all the filled wells and incubated again for 3 h. The plate was spun at 1500 rpm for 10 min followed by discarding approximately 80% of supernatant carefully. The volume of supernatant discarded was the same as the volume of DMSO added into the wells. The absorbance of each well was determined by a microplate reader at 550 nm after the purple crystal formazan fully dissolved in DMSO. Three independent experiments for both suspension and anchorage-dependant cell lines were conducted. The average of the absorbance values was used in the calculation of percentage of cell viability. The cytotoxicity index used was IC_50_, which is the concentration that yields 50% inhibition of the cell compared with the untreated control.

## 4. Conclusions

The stem bark of *Calophyllum soulattri* furnished one new diprenylated xanthone, phylattrin (**1**) together with five other xanthones, soulattrin (**2**), caloxanthone C (**3**), macluraxanthone (**4**), brasixanthone B (**5**) and trapezifolixanthone (**6**) and two common sterols, stigmasterol (**7**) and *β*-sitosterol (**8**). Compounds **1**–**7** exhibited moderate cytotoxicity towards several human cancer cell lines. Among the seven compounds tested, soulattrin (**2**) appears to have the best cytotoxic activity.
